# Detection and molecular characterisation of bovine *Enterovirus* in Brazil: four decades since the first report

**DOI:** 10.1017/S0950268818003394

**Published:** 2019-03-08

**Authors:** M. Candido, S. R. Almeida-Queiroz, M. G. Buzinaro, M. C. Livonesi, A. M. Fernandes, R. L. M. Sousa

**Affiliations:** 1 Department of Veterinary Medicine, Faculty of Animal Science and Food Engineering, University of São Paulo (FZEA/USP), Avenue Duque de Caxias Norte, 225, Jardim Elite, Pirassununga, São Paulo 13635-900, Brazil; 2 Department of Preventive Veterinary Medicine and Animal Reproduction, São Paulo State University (UNESP), Access Route Prof. Paulo Donato Castellani, Rural, Jaboticabal, São Paulo 14884-900, Brazil; 3 Department of Clinical Analysis, Faculty of Pharmacy, Alfenas Federal University (UNIFAL), Street Gabriel Monteiro da Silva, 700, Alfenas, Minas Gerais 37130-000, Brazil

**Keywords:** Animal viruses, bovine *enterovirus*, cattle diseases, molecular diagnostics

## Abstract

It is suggested that bovine enteroviruses (BEV) are involved in the aetiology of enteric infections, respiratory disease, reproductive disorders and infertility. In this study, bovine faecal samples collected in different Brazilian states were subjected to RNA extraction, reverse transcription-polymerase chain reaction analysis and partial sequencing of the 5′-terminal portion of BEV. One hundred and three samples were tested with an overall positivity of 14.5%. Phylogenetic analysis clustered these BEV Brazilian samples into the *Enterovirus F* clade. Our results bring an important update of the virus presence in Brazil and contribute to a better understanding of the distribution and characterisation of BEV in cattle.

Viruses of the Picornaviridae family of which enteroviruses (EVs) belong are non-enveloped and icosahedral viruses, measure 30–32 nm in diameter and present positive-sense, non-segmented RNA genome ranging in size from 6.7 to 10.1 kb containing a single long open reading frame. They infect humans and some animal species, and are taxonomically grouped into A-L *Enterovirus* species and A-C *Rhinovirus* species [1]. The classification of the EVs has been constantly changing with the discovery of new genetic sequences. The known bovine enteroviruses (BEV) belong to the species EV-E and -F; EV-E is subgrouped into four types and EV-F into six types. Details can be found in the Picornaviruses database [2].

Occasionally the virus causes serious and life-threatening diseases in humans and animals, but most EVs infections are subclinical [3, 4]. In bovine, EVs have been isolated from cattle with a wide range of clinical signs including enteric infections (high morbidity, diarrhoea and moderate mortality), respiratory disease (cough, fever and dyspnoea), reproductive disorders and infertility [5, 6]. EVs are important markers of faecal contamination by cattle, occurring more commonly in rural environments [7].

The first isolation of BEV occurred in the late 1950s [8]. The first and only work performed with these viruses in Brazil dates back more than 40 years. This work described the detection of BEV in two different Brazilian states (São Paulo and Pernambuco) through serological screening [9].

We present an important update of the presence of BEV in Brazil and also the first molecular characterisation of these viruses present in different Brazilian states.

From 15 July 2012 to 18 March 2016, 103 faecal samples were collected from the rectum of cattle (beef and dairy) in rural areas of the states of São Paulo, Minas Gerais, Goiás, Rio Grande do Sul, Paraná and Mato Grosso do Sul in Brazil. Of the studied animals, 36 (34.9%) had diarrhoea, 67 (65.0%) were aged <4 months and the majority (71 animals, 68.9%) were female. Seventy-four (71.8%) were raised under the feedlot system and 29 (28.1%) under free-range production conditions. Immediately after collection, the samples were kept in plastic bags at −4 °C until processing in the laboratory.

The RNA of samples was extracted using TRIzol™ Reagent (Invitrogen, USA). Reverse transcription was performed using the ImProm-II™ Reverse Transcription System (Promega, USA) and random primers (Invitrogen, USA), according to the manufacturer's instructions. Polymerase chain reaction (PCR) targeting a 183-bp fragment containing a part of the 5′-terminal portion of BEV [10] was done with a GoTaq™ Colorless Master Mix (Promega, USA), following the manufacturer's protocol, using the bovine β-actin gene as internal control [11]. The amplified products were gel extracted and sequenced directly on both strands with the same primers used in the PCR, in an automated ABI 3730 DNA Analyzer (Applied Biosystems, USA).

The results showed that 15 (14.5%) of the samples collected were positive for BEV including both calves and adult animals; five animals had diarrhoea, 11 were females and the majority of the positive samples were from dairy cattle (*n* = 13). Ten animals were from the state of São Paulo, four were from the state of Minas Gerais and one was from the state of Goiás.

Out of all PCR-positive samples, seven were selected and submitted to nucleotide sequencing because they represented the different cattle farms with BEV-PCR-positive results. However, only four sequences showed quality for subsequent analyses (two from state of São Paulo, one from state of Minas Gerais and one from state of Goiás). These four sequences shared >92.2% nt sequence identity when compared each other, 81.1%–86.6% nt identity when compared with *Enterovirus E* samples and 86.1%–92.8% nt identity when compared with *Enterovirus F* samples. Phylogenetic analysis of the partial sequences clustered these BEV Brazilian samples into the *Enterovirus F* clade (Fig. 1). Our results presented, after more than four decades, the occurrence of BEV in Brazil and the first phylogenetic characterisation of these viruses found in Brazilian cattle herds.

**Fig. 1.**
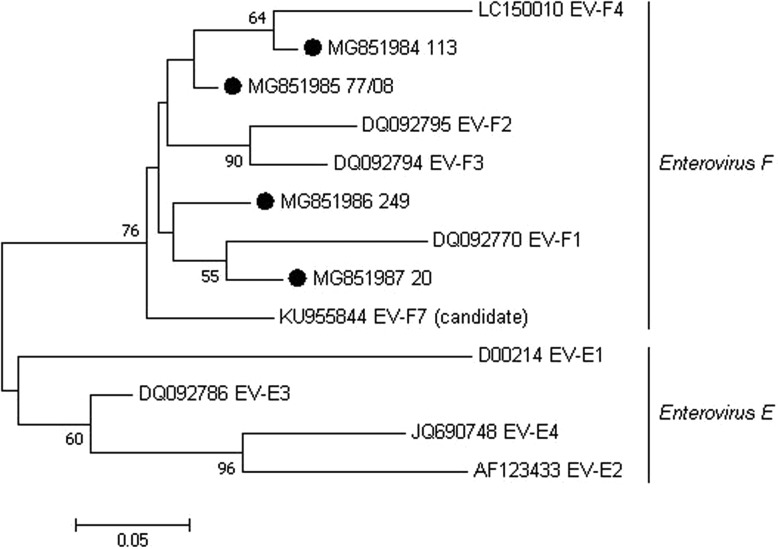
Maximum-likelihood unrooted phylogenetic relationships using a 183 bp-sequence of the 5′-terminal portion of BEV. Bootstrap values higher than 50% for 1000 pseudo-replicates are showed at the nodes. The sequences obtained in the present study are labelled with a filled circle. GenBank accession numbers and representative samples of the EV subgroups E and F are shown on the tree. The scale bar represents the phylogenetic distance among sequences.

BEV-infected cattle shed large amounts of virus in their faeces leading to environmental contamination, these animals constantly present mild to severe respiratory complications, varying degrees of diarrhoea and infertility [6].

Most of the samples used in this study were also used to investigate other viruses that cause gastroenteritis in cattle, and one of the samples was positive for both BEV and bovine Astrovirus [12]; two other samples were positive for both BEV and bovine *Kobuvirus* [13] and two samples were positive for both BEV and bovine *Picobirnavirus* [14]. It is interesting to note that in all cases of coinfection the animals were less than 8 months old. Some authors also reported the presence of different viruses in animal samples diagnosed with BEV [6, 15].

In this work we found BEV in animals without apparent diarrhoea and in adult animals, thus we cannot affirm that the viral strains detected in this study trigger diarrhoea in infected animals or are prevalent in younger animals.

This study is an important update of the presence of BEV in different Brazilian regions and describes the first partial molecular characterisation of these viruses in Brazil. Although it is not yet possible to define the degree of risk of the presence of the virus in the confirmed regions, there are several reports of serious problems involving BEV infection around the world, from gastroenteritis, respiratory problems to abortions [5, 6], which could lead to incalculable losses to the cattle farming sector.

Future studies using specific primers for the verification of BEV-E or even the complete genome of BEVs are required for the understanding of the diversity of strains and subgroups of these viruses in Brazil. In addition, the experimental infection of gnotobiotic cattle using BEV strains circulating in the different Brazilian states is necessary for a better understanding of the severity of the circulating viral strains in the country. Our results contribute to a better understanding of the distribution and characterisation of viral agents potentially associated with enteric, respiratory and reproductive infections in cattle.

## References

[1] Zell R (2017) ICTV virus taxonomy profile: *Picornaviridae*. Journal of General Virology 98, 2421–2422. 28884666 10.1099/jgv.0.000911 PMC5725991

[2] The Picornavirus Pages Available at http://www.picornaviridae.com/ (Accessed 4 September 2018).

[3] Blas-Machado U (2007) Fatal ulcerative and hemorrhagic typhlocolitis in a pregnant heifer associated with natural bovine enterovirus type-1 infection. Veterinary Pathology 44, 110–115. 17197635 10.1354/vp.44-1-110

[4] Palacios G and Oberste MS (2005) Enteroviruses as agents of emerging infectious diseases. Journal of NeuroVirology 11, 424–433. 16287683 10.1080/13550280591002531

[5] Ze-Li X (2014) Identification of a enterovirus E isolate from a cattle with respiratory diseases. Chinese Journal of Veterinary Science 34, 390–394.

[6] Zhang H (2014) Characterization of an enterovirus species E isolated from naturally infected bovine in China. Virus Research 191, 101–107. 25102330 10.1016/j.virusres.2014.07.032

[7] Blas-Machado U (2011) Pathogenesis of a *bovine enterovirus-*1 isolate in experimentally infected calves. Veterinary Pathology 48, 1075–1084. 21245281 10.1177/0300985810395728

[8] McFerran JB (1962) Bovine enteroviruses. Annals of the New York Academy of Sciences 101, 436–443.

[9] Linhares MI (1974) Bovine enterovirus antibodies in sera from São Paulo and Pernambuco States, Brazil. Arquivos do Instituto Biológico (Sao Paulo) 41, 1–4. 4466482

[10] Ley V, Higgins J and Fayer R (2002) Bovine enteroviruses as indicators of fecal contamination. Applied and Environmental Microbiology 68, 3455–3461. 12089028 10.1128/AEM.68.7.3455-3461.2002 PMC126779

[11] Renshaw RW, Ray R and Dubovi EJ (2000) Comparison of virus isolation and reverse transcription polymerase chain reaction assay for detection of bovine viral diarrhea virus in bulk milk tank samples. Journal of Veterinary Diagnostic Investigation 12, 184–186. 10730955 10.1177/104063870001200219

[12] Candido M (2015) Molecular detection and phylogenetic analysis of bovine astrovirus in Brazil. Archives of Virology 160, 1519–1525. 25797196 10.1007/s00705-015-2400-8

[13] Candido M (2017) Molecular characterization and genetic diversity of bovine *Kobuvirus*, Brazil. Virus Genes 53, 105–110. 27623839 10.1007/s11262-016-1391-1

[14] Navarro JO (2018) Genetic diversity of bovine *Picobirnavirus*, Brazil. Virus Genes 54, 724–728. 29987684 10.1007/s11262-018-1586-8

[15] Zhu L (2014) Identification of a novel enterovirus E isolates HY12 from cattle with severe respiratory and enteric diseases. PLoS One 9, 1–9. 10.1371/journal.pone.0097730 PMC4022658 24830424

